# Partial substitution, with their chelated complexes, of the inorganic zinc, copper and manganese in sow diets reduced the laminitic lesions in the claws and improved the morphometric characteristics of the hoof horn of sows from three Greek herds

**DOI:** 10.1186/s40813-016-0040-3

**Published:** 2016-09-07

**Authors:** Nikoleta Varagka, Marina Lisgara, Vassilis Skampardonis, Vassilis Psychas, Leonidas Leontides

**Affiliations:** 1 Department of Pathology, School of Veterinary Medicine, Aristotle University of Thessaloniki, 11 St. Voutira st., 54627 Thessaloniki, Greece; 2 Department of Epidemiology, Biostatistics and Economics of Animal Production, School of Veterinary Medicine, University of Thessaly, 224 Trikalon st., 43132 Karditsa, Greece

**Keywords:** Sows, Chelated minerals, Hoof horn quality

## Abstract

**Background:**

Hoof lesions in sows have been associated with lameness and poor hoof horn quality. The mechanical strength and quality of hoof horn is determined by the density and diameter of horn tubules, which were recently associated with the severity of lesions on the hoof wall of sows. Histologic changes that have previously been described in cases of bovine laminitis, have also been observed in the dermis and epidermis of the sows’ claws. Trace elements, particularly zinc, copper and manganese, occupy important roles as enzyme catalysts in the process of keratin synthesis which determines the quality and the integrity of the hoof epidermis. Therefore, the objective of this study was to investigate the effect of diet supplementation with chelated zinc, copper and manganese, partially substituting their inorganic form, on sow claw health and hoof horn quality assessed by macroscopic, histologic and morphometric examination.

**Results:**

Clinically, the total claw lesion score was significantly lower in claws of sows which received the “organic” diet compared to those of sows on the “inorganic” diet. Histologically, lamellar hyperplasia was the most frequently recorded change in the epidermis of the sows’ claws regardless of the diet’s mineral source. The claws of the sows which received the organic diet were more likely to have none or less histologic changes than at least one or more, respectively, compared to those of the sows on the “inorganic” diet. Morphometrically, the density and vertical and horizontal diameters of the horn tubules was significantly higher and smaller, respectively, in the hoof horn of sows which received the “organic” compared to those which received the “inorganic” source diet.

**Conclusions:**

Partial substitution of the inorganic zinc, copper and manganese in sows’ diet with their chelated complexes, provided a comparative advantage against a conventional, inorganic mineral source diet, at least under the conditions examined in the current study, in terms of macroscopic, histologic and morphometric criteria, characterizing the health and horn quality status of sows’ hooves.

## Background

Hoof lesions in sows have been associated with lameness [1–3] and poor hoof horn quality [4, 5]. The quality and integrity of the hoof epidermis essentially depends on normal keratinization. Keratinization is a complex process of differentiation of epidermal cells, characterized by a high rate of synthesis of keratin proteins as well as intercellular cementing substances [6] and finalized by the programmed death of the living epidermal cells (i.e., cornification) that turns the living epidermal cells into dead horn cells [7]. Because the epidermis is an avascular tissue, keratinocytes depend on the microvasculature of the corium for oxygen and nutrients supply by diffusion across the basement membrane [7]. When nutrient supply to keratinocytes is compromised or completely altered due to nutrient deficiency or inflammation of the dermis, inferior horn is produced [8]. In dairy cows the most common disorder within the horny tissues of the hoof is laminitis, which is the generic term for conditions in which the sensitive dermal structures between the pedal bone and the hoof horn are damaged [8, 9]. We have recently reported that the histologic changes which have previously been described in cases of bovine laminitis were also found in the dermis and epidermis of the claws of sows [5].

Laminitis is associated with impaired synthesis or disturbed chemical binding of keratins, the structural proteins of the hoof, with resultant deterioration of the macromolecular organization that gives the horn mechanical strength [10]. The mechanical strength is determined by the density and diameter of horn tubules [7, 11, 12]. Each horn tubule consists of an outer cortex originating from the living epidermis located around the dermal papilla and an inner medulla originating from the epidermis over the tip of the papilla [7]. Horn quality is the product of horn morphometric characteristics (i.e., the diameter and density of tubules and the ratio between cortex and medulla), hoof shape and the anatomy and physiology of the dermis which provides oxygen and nutrients to keratinocytes and modulates and controls cell differentiation in the epidermis including keratin formation [8, 13]. We recently reported, after morphometric examination of claws from sows fed with conventional, inorganic sources of minerals, that the diameter and density of horn tubules are positively and negatively, respectively, associated with the severity of lesions in the hoof wall, suggesting an inter-relationship of decreased hoof horn quality in sows’ claws with lesions on the hoof horn [5].

During the process of keratinization, the epidermal cells require sufficient and balanced supply of nutrients [7]. The trace elements zinc, copper and manganese play important roles as enzyme catalysts in keratin synthesis [14]. A reduction in the incidence of digital disorders associated with laminitis was observed in cows fed a combination of organic trace minerals [15]. Zinc has been identified as the key micromineral in the process of keratinization [7, 16] and several studies have shown that zinc methionine improves hoof integrity in cattle [17–19]. The role of copper in keratin synthesis is through the enzyme thiol oxidase, a key enzyme necessary for the cross-linking of keratin filaments within the keratinocyte [20]. This process provides structural strength and rigidity to the keratinized cell matrix [8, 21], making it more resistant to mechanical and physical forces [22]. Lastly, manganese is involved in a number of enzyme systems and is required for cartilage and collagen formation and bone growth [23].

The valence state of the mineral and its molecular form (inorganic or organic) in the diet play an important role in the bioavailability of trace minerals [24]. These specific characteristics of the mineral may be responsible for the complexes they form with other ingredients in the gut, which may either hamper or assist the mucosal absorption, transport and/or metabolism of the mineral in target tissues [24]. When conventional inorganic oxides and sulfates (e.g., ZnO, CuSO_4_) in feed break down in the stomach, the released ions may either remain soluble in the gut by interacting with components, such as amino acids and carbohydrates or interact with water molecules to form an insoluble hydroxyl-metal species or bind to ligands with phytate and form low soluble and unabsorbable salts [25]. Chelated mineral complexes, which are minerals joined with an organic ligand such as a protein or a specific amino acid, are more stable in the digestive tract and protected from forming complexes with other dietary components that would otherwise inhibit absorption [25–27]. Therefore, by utilizing the intestinal uptake processes of amino acids [28], organic Zn, Cu and Mn, which are structurally and functionally important for keratinization, are absorbed by the intestinal epithelium more easily compared to inorganic ones [29].

Thus, the objective of this study was to investigate the effect of diet supplementation with chelated zinc, copper and manganese, partially substituting their inorganic form, on claw health and hoof horn quality, assessed by macroscopic, histologic and morphometric examination, in sows from three conventional farrow-to-finish Greek herds.

## Methods

### Study design

Sampled sows, which were culled at weaning, originated from three Greek, indoor, farrow-to-finish herds with 330 (A), 160 (B) and 800 (C) sows, respectively, with Danbred (A, B) and Hermitage (C) genetics. During gestation sows were loose housed in static groups of eight to 12, with free-access to individual feed troughs in non-locking stalls, on combinations of concrete and slatted flooring. Pen design and flooring was in accordance to the requirements of the EU Directive 2001/88/EC for loose housing of gestating sows. All herds operated on weekly farrowing schedules. For participation in the study, the only criterion was the owner’s written consent. The health status of the sows’ feet was not considered for herd selection.

One front and the opposite rear foot from 153 sows (612 claws from 306 feet), culled at weaning, were collected, altering selection between left or right front foot of successively sampled sows, from three Greek slaughterhouses, which operate in accordance with the European legislation (93/119/EC) for slaughtering animals without unnecessary suffering, during an eighteen-month period. The study was divided in two stages. Sows in the study herds were similarly fed in both stages, with the exception of the source of minerals: they were offered, from exit from the farrowing facilities until service, a total of 4.0-4.5 kg daily of gestation feed. Thereafter, the daily amount of feed was 2.6–2.8 kg until 90 days and 3.2–3.5 kg from day 91 to 107 of gestation. The feed contained 12.6–12.8 MJ metabolizable energy (ME), 14.0–14.4 % crude protein, 6.2–6.4 g of digestible lysine, 15000 IU of vitamin A, 130 mg of vitamin E, 8.2–8.5 g of calcium (Ca), 5.2–5.5 g of phosphorus (P) and 0.45 mg of biotin per kg and was given either in one meal at 0700 h (Herd B and C) or split in half and offered in two meals at 0700 and 1600 h (Herd A). One week before the expected farrowing, sows were transferred to the lactation facilities where feeding was restricted until five days after farrowing and then were offered ad libitum typical lactation diets containing 13.5 MJ ME/kg dry matter and 16.5–17.0 % crude protein, 7.5–7.7 g of digestible lysine, 17000 IU of vitamin A, 180 mg of vitamin E, 8.9–9.2 g of Ca, 5.7–6.0 g of P and 0.45 mg of biotin. The first stage, which was conducted during the first six months of the study period, included the collection of feet from sows which were raised and fed throughout their life, in all study herds, with diets containing only inorganic sources of minerals (Inorganic Diet: ID). The ID, which was in meal form, contained in total 125 mg/kg feed Zn from ZnO, 15 mg/kg Cu from CuSO4 and 40 mg/kg Mn from MnO. At this stage, 156 feet (312 claws) from 78 sampled sows were collected; 25 sows (32.0 %) were from herd A, 15 sows (19.2 %) were from herd B and 38 sows (48.7 %) were from herd C. Parity of these sows ranged from one to ten (median 7 parity), two to nine (median 4 parity), and two to eight (median 6 parity) for herds A, B and C, respectively. The second stage corresponded to the collection of feet from sows, from the same herds, after the supplementation of their diets with chelated trace minerals (Supplemented Diet: SD). The chelated trace minerals were supplied from commercially available chelated mineral sources (Zinpro Performance Minerals®, Availa®Sow), substituting partially their inorganic form (organic form 45 of the total 125 ppm of Zn, 14 of the total 15 ppm of Cu and 25 of the total 40 ppm of Mn). The switch to the SD was applied within the same month in all three farms. This intervention was the first occasion on which sows in these herds received a diet with organic mineral sources. Feet were collected from sows which were culled after being fed the SD for at least one or two complete reproductive cycles; each cycle included one full-term gestation and four weeks lactation. The duration of one cycle may be considered minimum for complete hoof horn regeneration [30] because the sows’ claw, which on average is 5 cm long, grows with a mean rate of about 6.3 mm and wears with a mean rate of about 5.1 mm per month [31]. During this second phase of the study we collected 150 feet (300 claws) from 75 sows; 27 (36.0 %) sows were from herd A, 16 (21.3 %) sows were from herd B and 32 (42.7 %) sows were from herd C. Parities ranged from one to nine (median 6 parity), one to eight (median 5.5 parity) and one to nine (median 5 parity) for herds A, B and C, respectively.

The technicians which collected the feet at slaughter were blinded to the purpose of the study and had not been trained to recognize claw lesions, thus, reducing potential bias towards selection of claws with more lesions. In addition to feet collection, they recorded for each sampled sow its identification number and parity (retrieved from each herd’s managerial database). The collected feet and the ear tag of the sow were placed in the same plastic bag. All bags were placed in polystyrene cooling boxes and transferred, within one day, to the Aristotle University of Thessaloniki, School of Veterinary Medicine, Department of Pathology. On the day of arrival, claws were macroscopically examined, sectioned, and fixed in 10 % neutral buffered formaldehyde in order for their histologic and morphometric examination to be carried out.

### Macroscopic examination

The medial and lateral claws of the collected 306 front and rear feet, were macroscopically examined for lesions and scored by one of the authors (VP). The scoring system applied has been described in detail [3]. Briefly, for each claw, five anatomical sites were examined: the heel (soft keratinized epidermis on the ventral surface of the claw towards the posterior end); the sole (hard keratinized epidermis anterior to the heel on the ventral surface of the claw including the junction between heel and sole); the white line (junction between sole and wall), the wall (hard keratinized epidermis on the dorsal surface of the claw); and the coronary band. The above anatomical sites of the claw were examined for the presence of cracks, erosions, ulcers, bruises, separation along the white line, and hyper-keratinization. The evaluation of the anatomical sites of the claw involved a severity scale ranging from 0 to 2, where score 0 was assigned to claw sites with no lesions or very small superficial ones. For the sole, score 1 was given to hooves with erosions and/or superficial heel-sole separation and 2 to hooves with ulcers and/or severe heel-sole separation. For the heel, score 1 was given to hooves with hyper-keratinization and erosions and 2 to hooves with hyper-keratinization and ulcers. For the white line score 1 was assigned to hooves with superficial separation and 2 to hooves with deep separation. For the wall, the score was 1 when cracks often accompanied by bruises were observed and 2 when deep cracks were noted. For the coronary band the score ranged from 0 to 1 (0 = no lesions, 1 = lesions of any kind, edema, hemorrhage and/or necrosis).

### Histologic examination

The technique for histologic slice collection has been described in detail [5]. Briefly, a slice (width 0.5 cm) was cut with a band saw perpendicular to the dorsal wall of each of the 612 claws (153 sows × 2 feet × 2 claws) that had been previously collected. The central point of the slice was at the midpoint between the coronary band and the weight-bearing area of the wall, at the junction of the wall and sole. From the extracted slice of tissue, a sample (0.5 cm × 0.5 cm × 0.5 cm) was cut from the wall segment of the claw. The isolated tissue sample, which consisted of dermis and epidermis, was separated from the underlying bone by a scalpel incision through the dermis as close to the pedal bone as possible.

Samples were fixed for one week, then dehydrated through graded concentrations of ethanol and xylene using an automatic tissue-processing machine (Shandon 2LE tissue processor; Shandon Southern Products Ltd, Astmoor, Runcorn, Cheshire, England), and embedded in paraffin wax. A sledge microtome was used to cut 5-μm horizontal sections from each sample. The sections were stained with hematoxylin and eosin and examined for pathologic changes indicative of laminitis [5], under a light microscope at × 10, ×20, and × 40 magnifications. The pathologic changes identified included lamellar hyperplasia, the presence of white blood cells, hyperemia, hemorrhage, edema, and necrosis of the dermis. Therefore, we recorded the number of suprabasal cell layers along the cornified part of the epidermal lamellae. One or two layers were classified as normal, whereas three or more were classified as increased (lamellar hyperplasia). In normal claws, the capillaries of the dermis appear small and their lumina are usually empty. Reactive hyperemia is the first physiologic event of acute laminitis [32]. In this study, hyperemia was recorded when the vessels were filled with red blood cells up to the tips of the lamellae. Hemorrhage was noted if blood components (plasma and hemosiderin) were found inside tubules. Edema was noted if normal tissue components were spread apart, giving the tissue a less dense appearance. Necrosis was noted when pyknosis or karyolysis of several cells were observed. Pyknosis is defined as the shriveling of the nucleus of the dying cell into an intensely basophilic uniform mass and karyolysis is defined as the gradual histological disappearance of the nucleus and its macromolecular contents, which literally fade away to nothing [33].

### Morphometric examination

Due to laboratory limitations at the first stage of the study, a convenience sample of slices from the lateral claw of the rear foot of 52 sows, out of the 78 sampled sows, were used to evaluate the morphological features of the horn tubules of the claws, whereas at the second stage of the study, slices from the lateral claw of the rear foot of all 75 sampled sows were morphometrically examined. Morphometric evaluation of the slices of the claws, collected during the first stage, was conducted in 20 claws without wall lesions (score 0), 21 claws with bruises or superficial cracks on the wall (score 1), and 11 claws with deep wall cracks (score 2). From claws collected during the second stage, the morphometric evaluation was done on slices from 54 claws without wall lesions (score 0), 17 claws with bruises or superficial cracks on the wall (score 1), and 4 claws with deep wall cracks (score 2). We opted to perform the morphometric examination on slices from the lateral claws of the rear feet because they are most commonly and severely affected [2, 5, 34].

Tubular architecture of sows’ claws comprised three zones of morphologically different tubules: an outer zone with flattened tubules (zone A), an intermediate zone with round to oval tubules (zone B), and an inner zone with tiny horn tubules (zone C) [5], resembling that of equine and bovine hooves [35–37]. Two representative fields magnified × 10 and two magnified × 20 in each zone of each sample were captured using a Nikon eclipse 50i microscope and a Nikon DS-5 M-L1 digital camera (Nikon Instruments Inc, Melville, New York). At the lower magnification, the tubules in each image were counted using the cell count plug-in of the ImageJ image processing and analysis program (NIH, Bethesda, Maryland), and at the higher magnification, the vertical and horizontal diameter of three representative tubules were measured.

The histologic and morphometric evaluations were performed by one of the authors (NV), who was blinded to the results of the macroscopic examination.

### Statistical analysis

All statistical analyses were performed using Stata 13.1 (Stata Statistical Software, College Station, Texas).

#### Macroscopic examination

For each claw site, the frequency of lesions and their severity was calculated by diet mineral source (ID and SD), foot (front and rear) and toe (medial and lateral) location. For each claw or foot, the total lesion score, which could range from 0 to 9 or from 0 to 18, respectively, was calculated as the sum of the scores of the five examined anatomical sites for either claw or both claws, respectively. Two sample *t-*tests were used to compare the mean total lesion scores of medial and lateral claws on the same foot and the mean total lesion score of front and rear foot, between the sows that received the ID and the SD.

The total lesion score of each claw was associated with diet mineral source in a multi-level linear regression model in GLAMM [38, 39]. In this model, the total lesion score was the dependent variable, whereas the source of the minerals supplementing the offered diets, the foot, the toe, the sow’s parity and the farm of sow’s origin were the independent variables. The latter two variables were forced in the models in order to control for their likely confounding effects. Furthermore, a random-effect term for sow and a random-effect term for foot nested within sow were included in order to account for the multiple measurements on the same animal and on claws of the same foot. After initial fit of the model to the data all possible two-way interactions between the fixed effects for diet mineral source, foot and toe were created and then offered one-by-one to the initial model and evaluated for significance at the 5 % level. The final model contained the fixed-effects for diet mineral source, foot and toe, and the random-effects.

#### Histologic examination

For each foot and toe, the frequency of pathological changes recorded in tissue samples of dermis and epidermis of claw sections from the midpoint of the dorsal wall was calculated, both for sows that received the ID and the SD. Pearson’s χ^*2*^ tests were used to compare the frequency of the pathological changes of the lateral and the medial toes on the same foot (front or rear), between the sows that were on ID and the sows that were on SD. The frequency of sows with at least one recorded pathological change on any claw, for each type of histologic change examined, between those on the ID and the SD, was also compared with Pearson’s χ^*2*^ test.

The existence of lamellar hyperplasia, which was the most frequently recorded pathological change, was associated with the source of minerals in the diets (ID and SD) in a multi-level linear regression model in GLAMM [38, 39]. In this model, the presence of hyperplasia at claw level was the dependent variable, whereas the source of the minerals supplementing the offered diets, the respective total claw lesion score, the foot, the toe, the sow’s parity and the farm of sow’s origin were the independent variables. A random-effect term for sow and a random-effect term for foot nested within sow were also included in order to account for the multiple measurements on the same animal and foot. Similar analytical models were not used for the other pathological changes because they were either infrequently recorded (necrosis, hemorrhage, hyperemia, presence of white blood cells) or frequently recorded but usually co-existing with lamellar hyperplasia (edema).

Additionally, the number of pathologic changes recorded in each claw was associated with the source of minerals in the offered diets in a multi-level ordinal regression model in GLAMM. In this model, the number of recorded pathologic changes, which ranged from 0 to 5, was the dependent variable, whereas the source of the minerals supplementing the offered diets, the respective total claw lesion score, the foot, the toe, the sow’s parity and the farm of sow’s origin were the independent variables. Total claw lesion score was included in the model, because of its association with the presence and frequency of specific histologic changes in the dermis and epidermis of sow’s claw [5]. Furthermore, a random-effect term for sow and a random-effect term for foot nested within sow were included in order to account for the multiple measurements on the same animal and foot. The proportional odds assumption for the fitted ordinal regression model was verified graphically, by assessing the plots of the resulting empirical cumulative logit functions of each of the dependent variables considered. After initial fit of the model to the data all possible two-way interactions between the fixed effects for diet mineral source, foot and toe were created and then offered one-by-one to the initial model and evaluated for significance at the 5 % level. The final model contained the fixed-effects for diet mineral source, foot and toe, the significant two-way interactions between the fixed effects and the random-effects.

#### Morphometric examination

The density and the horizontal and vertical diameters of the horn tubules were calculated by wall macroscopic lesion score, zone and diet mineral source (ID and SD). Two sample *t-*tests were used to compare the mean tubular density and the mean horizontal and vertical diameter by wall macroscopic score and zone, between the sows which were offered the ID and the SD. Then, the three measurements were associated with diet mineral source in three multi-level linear regression models in GLAMM [38, 39]. In these models, diet mineral source, wall lesion score, zone, sow parity and farm of sow’s origin were included as fixed-effect terms, the field as random-effect term nested within zone, and the sow as random-effect term. The random effect terms were included in order to account for the multiple measurements on the same field and animal. After initial fit of the model to the data all possible two-way interactions between the fixed effects for diet mineral source, wall lesion score and zone were created, and offered one-by-one to the initial models and evaluated for significance at the 5 % level. The final models contained the fixed-effects for diet mineral source, wall lesion score and zone, and the significant two-way interactions between the fixed effects and the random-effects.

## Results and discussion

### Results of the macroscopic examination

The frequency of lesions recorded and their severity scale, by site and toe as well as the mean of the total lesion score by foot, for the 78 sampled sows on the ID, and for the 75 sampled sows on the SD are presented in Table 1. The most frequently observed lesions were those located on the heel, the wall and the white line, for both groups of sows. Specifically, for sows which received the ID, 158 (50.6 %), 104 (33.3 %) and 85 (27.2 %) claws out of the 312 examined, had at least one lesion on the heel, the wall and the white line, respectively, whereas for sows received the SD, 80 (26.7 %), 71 (23.7 %) and 42 (14.0 %) claws out of the 300 examined had at least one lesion on the heel, the wall and the white line, respectively. The total lesion score, adjusting for foot and toe location, was significantly lower (*P* < 0.001, Coef = − 0.85, 95 % CI: −1.13, −0.58) on claws of sows that received the SD compared to those of sows on the ID. The total lesion score was, additionally, higher on rear (*P* = 0.001, Coef = 0.33, 95 % CI: 0.14, 0.52) compared to front feet and on lateral (*P* < 0.001, Coef = 0.80, 95 % CI: 0.62, 0.98) compared to medial toes.

**Table 1:** Frequency and mean total score of claw lesions for culled sows that received the ID and the SD

Foot	Toe	Score	Dietary status	Sole	Heel	White line	Wall	Coronary band	Mean total score	
Front	Lateral	0	ID	78.21	48.72	71.80	66.67	98.72	5.07^1a^	2.73^2a^
			SD	89.33	74.67	85.33	74.67	94.67	3.47^1b^	1.88^2b^
		1	ID	20.51	24.36	21.79	30.77	1.28		
			SD	10.67	21.33	14.67	24.00	5.33		
		2	ID	1.28	26.92	6.41	2.56	NA		
			SD	0.00	4.00	0.00	1.33	NA		
	Medial	0	ID	83.33	61.54	74.36	69.23	100		2.34^3a^
			SD	94.67	81.33	92.00	77.34	97.33		1.59^3b^
		1	ID	16.67	23.08	23.08	25.64	0.00		
			SD	5.33	18.67	8.00	21.33	2.67		
		2	ID	0.00	15.38	2.56	5.13	NA		
			SD	0.00	0.00	0.00	1.33	NA		
Rear	Lateral	0	ID	65.39	23.08	65.39	58.98	98.72	5.80^4a^	3.60^5a^
			SD	72.00	46.67	73.33	72.00	100	4.06^4b^	2.59^5b^
		1	ID	25.64	29.49	26.92	33.33	1.28		
			SD	28.00	38.67	24.00	22.67	0.00		
		2	ID	8.97	47.43	7.69	7.69	NA		
			SD	0.00	14.66	2.67	5.33	NA		
	Medial	0	ID	85.90	64.10	79.49	71.79	98.72		2.20^6a^
			SD	92.00	90.67	93.33	81.33	100		1.47^6b^
		1	ID	12.82	26.92	15.38	23.08	1.28		
			SD	8.00	8.00	6.67	16.00	0.00		
		2	ID	1.28	8.98	5.13	5.13	NA		
			SD	0.00	1.33	0.00	2.67	NA		

The frequency (%) of lesions and the mean of the total lesion score of 312 claws from 78 sows that were on diets only with inorganic sources of Zn, Cu and Mn (Inorganic Diet: ID) until they were culled at weaning, and the frequency and the mean of the total lesion score of 300 claws from 75 sows, which were on diets with organic sources of Zn, Cu and Mn as a partial substitution of their total inorganic form (Supplemented Diet: SD), for one or two gestations until they were culled at weaning, from three Greek herds. The frequency of lesions is presented by anatomical site, severity score and by foot (front or rear) and toe (medial or lateral) location, and the mean of the total lesion score is presented by foot and toe.

^1, 2, 3, 4, 5, 6^Numbers in the superscript define the compared pairs of mean total lesion scores between the two mineral source diets.1 = front feet, 2 = lateral claws of front foot, 3 = medial claws of front foot, 4 = rear feet, 5 = lateral claws of rear foot and 6 = medial claws of rear foot.

^a,b^Pairs of mean total lesion scores with different lower case superscript letters are significant different (*P* < 0.05; Two sample *t* test).

*NA* not applicable because the lesions score of CB ranged from 0 to 1.

### Results of the histologic examination

The frequency of histologic changes recorded by foot and toe for the 78 sampled sows which were on the ID and for the 75 sampled sows which were on the SD until they were culled, are presented in Table 2. Lamellar hyperplasia, leading to lamellar widening, was the most frequently recorded change in the epidermis of 92 of 312 (29.5 %) examined claws in 54 of 78 (69.2 %) sows that received the ID and of 65 of 300 (21.7 %) examined claws in 39 of 75 (52.0 %) sows that received the SD. Among claws without lesions, one or more histopathologic changes were recorded in 35 of 93 (37.6 %) claws collected from sows which received the ID and in 36 of 142 (25.4 %) claws collected from sows which received the SD. Hyperplasia was noted in 21 of 93 (22.6 %) and 24 of 142 (16.9 %) examined claws without lesions and in 71 of 219 (32.4 %) and 41 of 158 (25.9 %) examined claws with lesions which were collected from sows which received the ID and the SD, respectively. Widening and disruption of the dermal lamellae due to edema was noted in the dermis of 66 of 312 (21.2 %) claws in 46 of 78 (59.0 %) sows which received the ID and in 58 of 300 (19.3 %) claws in 43 of 75 (57.3 %) sows which received the SD. White blood cells were found in the dermis of 38 of 312 (12.2 %) examined claws in 33 of 78 (42.3 %) sows which were on the ID and of 43 of 300 (14.3 %) examined claws in 34 of 75 (45.3 %) sows which were on the SD. Densely stained material and hemosiderin were found inside tubules of 36 of 312 (11.5 %) and of 7 of 300 (2.3 %) examined claws in 28 of 78 (35.9 %) and 6 of 75 (8 %) sows which were offered the ID and the SD, respectively. Extensive necrosis in the dermis and epidermis was noted in 14 of 312 (4.5 %) and in 9 of 300 (3.0 %) examined claws in 12 of 78 (15.4 %) and in 9 of 75 (12.0 %) sows which were on the ID and those which were on the SD, respectively. In these cases, hyperplasia was not found. Hyperemia in the dermis was observed in only 4 of 312 (1.3 %) and in 12 of 300 (4.0 %) claws in 3 of 78 (3.8 %) and 8 of 75 (10.7 %) sows that were offered the ID and the SD, respectively.

**Table 2:** Frequency of histologic changes for the claws of culled sows that received the ID and the SD

Foot	Toe	Diet	Lamellar hyperplasia (%)	Edema(%)	Necrosis (%)	Hemorrhage (%)	Hyperemia (%)	White blood cells (%)
Front	Medial	ID	20^a^(25.64)	14^a^(17.95)	2^a^(2.56)	6^a^(7.69)	2^a^(2.56)	9^a^(11.54)
		SD	13^a^(17.33)	13^a^(17.33)	0^a^(0.00)	2^a^(2.66)	3^a^(4.00)	7^a^(9.33)
	Lateral	ID	22^a^(28.21)	20^a^(25.64)	5^a^(6.41)	9^a^(11.54)	0^a^(0.00)	5^a^(6.41)
		SD	14^a^(18.66)	14^a^(18.66)	2^a^(2.66)	1^b^(1.33)	4^b^(5.33)	9^a^(12.00)
Rear	Medial	ID	20^a^(25.64)	12^a^(15.38)	3^a^(3.85)	7^a^(8.97)	1^a^(1.28)	7^a^(8.97)
		SD	11^a^(14.66)	8^a^(10.66)	1^a^(1.33)	1^b^(1.33)	3^a^(4.00)	6^a^(8.00)
	Lateral	ID	30^a^(38.46)	20^a^(25.64)	4^a^(5.13)	14^a^(17.95)	1^a^(1.28)	17^a^(21.79)
		SD	27^a^(36.00)	23^a^(30.66)	6^a^(8.00)	3^b^(4.00)	2^a^(2.66)	21^a^(28.00)

Number and frequency (%) of histologic changes recorded in tissue samples of dermis and epidermis of 312 claws from 78 sows that were on diets with inorganic sources of Zn, Cu and Mn (Inorganic Diet: ID) until they were culled at weaning and of 300 claws from 75 sows which were on diets with organic sources of Zn, Cu and Mn (Supplemented Diet: SD) for one or two gestations until they were culled at weaning, from three Greek herds. The frequencies are presented by foot (front or rear) and toe (medial or lateral). The tissue samples were taken from claw sections from the midpoint of the dorsal wall.

^a,b^Different lower case superscript letters indicate significant difference between the ID and the SD, for each claw of the front and rear feet separately (*P* < 0.05; Pearson’s χ^*2*^ test).

The proportion of sows with hyperplasia in the epidermis of at least one of the examined claws was significantly lower (*P* = 0.029) for the sows that received the SD compared to those that received the ID. Similarly, the proportion of sows with densely stained material and hemosiderin inside tubules (hemorrhage) in at least one of the examined claws was significantly lower (*P* < 0.001) for the former compared to the latter sows. However, there was no statistically significant difference (*P* = 0.30, 95 % CI: −0.87, 0.27) in the probability of occurrence of hyperplasia between the claws of sows that received the two sources of minerals supplementing the offered diets. The probability of occurrence of hyperplasia, regardless of the diet’s mineral source, was 1.26 times higher for one unit increase of the total claw lesion score (*P* = 0.004, 95 % CI: 1.07, 1.49).

The odds ratio of higher categories of number of histologic changes (i.e., more histologic changes) compared to lower categories of number of histologic changes (i.e., none or fewer histologic changes) per unit of total lesion score was 1.39 (95 % CI: 1.18, 1.64) for sows fed the ID, while for sows fed the SD was 0.05 (95 % CI: 0.009, 0.28). More explicitly, for one unit increase of total lesion score, the odds of having at least one or more histologic changes recorded compared to have none or less histologic changes recorded were 1.39 times greater for claws of sows that received the ID, whereas it was 0.05 times lower for the claws of sows that received the SD.

### Results of the morphometric examination

The density and the horizontal and vertical diameter of the horn tubules are summarized by wall lesion score and zone, for the sampled sows that received the ID and the SD, in Table 3.

**Table 3:** Mean tubular density and horizontal and vertical diameter of the hoof wall of claws from culled sows that received the ID and the SD

Morphometric characteristic	Diet	Score 0			Score 1			Score 2		
		Zone A	Zone B	Zone C	Zone A	Zone B	Zone C	Zone A	Zone B	Zone C
Tubular density (±Sd)	ID	65.18^a^(±10.27)	61.95^a^(±7.55)	65.63^a^(±13.44)	64.38^a^(±13.44)	61.17^a^(±12.67)	60.55^a^(±17.97)	60.55^a^(±11.93)	57.00^a^(±12.82)	49.32^a^(±14.43)
	SD	71.94^b^(±7.67)	65.86^b^(±6.46)	57.97^b^(±6.60)	72^b^(±6.31)	63.08^a^(±8.35)	54^b^(±6.95)	56.75^a^(±12.03)	49.75^a^(±8.53)	50.63^a^(±9.98)
Horizontal diameter (±Sd)	ID	51.87^a^(±17.77)	40.30^a^(±16.21)	39.00^a^(±12.43)	50.14^a^(±17.14)	50.00^a^(±20.00)	40.95^a^(±13.14)	65.47^a^(±36.31)	68.10^a^(±40.18)	40.33^a^(±12.95)
	SD	42.93^b^(±7.34)	38.11^b^(±6.43)	31.11^b^(±5.43)	47.97^a^(±14.56)	43.98^b^(±8.14)	31.78^b^(±6.77)	64.41^a^(±23.25)	41.92^b^(±6.87)	32.01^b^(±5.90)
Vertical diameter (±Sd)	ID	17.15^a^(±5.26)	19.94^a^(±5.28)	20.34^a^(±5.00)	16.42^a^(±4.71)	22.06^a^(±6.21)	21.10^a^(±7.61)	20.88^a^(±11.02)	23.30^a^(±11.25)	18.86^a^(±7.72)
	SD	11.17^b^(±2.21)	15.04^b^(±3.16)	13.79^b^(±2.86)	15.21^b^(±3.88)	19.04^b^(±5.38)	14.47^b^(±3.91)	17.84^a^(±4.57)	20.07^a^(±4.60)	15.60^a^(±3.95)

The mean tubular density (number of horn tubules per field at × 10 magnification) and the mean horizontal and vertical diameter of horn tubules (in μm), and their standard deviations by wall lesion score and zone, as observed and measured in histologic slices from the wall of the lateral claw of the rear foot of 52 sows that were on diets with inorganic sources of Zn, Cu and Mn (Inorganic Diet: ID) until they were culled at weaning, and of 75 sows, which were on diets with organic sources of Zn, Cu and Mn (Supplemented Diet: SD) for one or two gestations until they were culled at weaning. Slices for morphometric evaluation were selected from the lateral claws of the rear feet because they were most commonly and severely affected. Three zones of morphologically different tubules were identified: Zone A, an outer zone with flattened tubules; Zone B, an intermediate zone with round to oval tubules; and Zone C, an inner zone with tiny horn tubules. Wall score 0 = no lesions; score 1 = bruising or superficial cracks; and score 2 = deep cracks

^a,b^Different lowercase superscript letters indicate significant difference between the ID and SD, for each zone and lesion score separately (*P* < 0.05; Pearson’s χ^*2*^ test)

The density of the tubules was higher (*P* = 0.002) by 4.97 tubules (95 % CI: 1.85, 8.11) per field, on average, among claws of animals that received the SD compared to those which received the ID in zone A. Higher density of horn tubules in the outer zone has been associated with increased hardness of the hoof horn and less claw lesions [5, 8]. Tubular density did not differ between claws of the two groups of animals (*P* = 0.35, 95 % CI: −1.64, 4.62) in zone B, whereas density was smaller (*P* < 0.001) by 5.74 tubules (95 % CI: −8.87, −2.61) per field, on average, among claws of animals that received the SD compared to those of sows on the ID, in zone C. The presence of horn tubules in the inner zone was described as indicator of laminitis in both pigs [40] and horses [41], because of the higher activity of this region in tubular production from the epidermal lamellae [42, 43]. Density of tubules was also smaller in claws with wall lesion score 1 (*P* = 0.028) and 2 (*P* < 0.001) compared to claws with wall lesion score 0, by 3.25 (95 % CI: −6.14, −0.35) and 10.78 (95 % CI: −14.79, −6.78) horn tubules, respectively.

The horizontal diameter of the tubules was smaller in claws of animals that received the SD compared to those which received the ID (*P* < 0.001) by 5.01 μm, on average (95 % CI: −7.00, −3.05). Additionally, the horizontal diameter was smaller in tubules of zone B (*P* < 0.001) and zone C (*P* < 0.001) compared to those of zone A, by 4.92 μm (95 % CI: −6.18, −3.65) and 13.94 μm (95 % CI: −15.2, −12.66) respectively, whereas it was larger in claws with wall lesion score 2 (*P* = 0.001) and 3 (*P* < 0.001) compared to claws with wall lesion score 1, by 4.22 μm (95 % CI: 1.66, 6.78) and 13.56 μm (95 % CI: 11.17, 15.95), respectively. Lastly, the vertical diameter of the tubules was smaller in claws of animals that received the SD compared to those which received the ID (*P* < 0.001) by 4.15 μm, on average (95 % CI: −5.27, −3.03). Moreover, the vertical diameter was larger in tubules of zone B (*P* < 0.001) and zone C (*P* < 0.001) compared to those of zone A, by 3.81 μm (95 % CI: 3.35, 4.27) and 2.04 μm (95 % CI: 1.58, 2.50) respectively, whereas it was also larger in claws with wall lesion score 2 (*P* < 0.001) and 3 (*P* < 0.001) compared to claws with wall lesion score 1, by 2.05 (95 % CI: 1.03, 3.07) and 3.43 (95 % CI: 2.15, 4.70) μm, respectively.

## Discussion

Claw lesions are extremely frequent and associated with locomotor disorders [2, 3, 44], compromised longevity [45, 46] and reduced productivity of sows [47–49]. Therefore, prevention is of utmost importance both for maintenance and improvement of the breeding herd’s potential and profitability of the farm. Prevention, which should be based on solid comprehension of the pathogenesis and determination of the causal and predisposing factors of claw lesions, should include strategies that discourage the development of claw lesions and/or mitigate their frequency and severity. Recently, we reported [5] that the histologic changes previously described in cases of bovine and equine laminitis can also be observed in dermis and epidermis of the claws of sows and may lead to production of low-quality hoof horn. These histologic changes may be regarded as the causes and not the consequences of sows’ claw lesions, since they were more likely to be present in claws with higher severity score (i.e., total claw lesion score) and were further observed in claws without clinically evident lesions [5]. Therefore, any measures, attempting to reduce the frequency and severity of claw lesions in sows, should, primarily, target towards the reduction of the incidence of histologic changes in the claw and the production of high-quality hoof horn.

In this study we macroscopically examined and scored lesions of the claws of one front and the opposite rear foot from 78 sows that were on ID until they were culled at weaning and from 75 sows, which were offered SD for one or two gestations until they were culled at weaning, from three herds. Lesion scores were recorded for five anatomical sites of the claw, namely the wall, the sole, the white line, the heel and the coronary band. The most frequently affected claw sites, in accordance with others [2, 5, 30, 34, 44, 50], were the heel, the wall and the white line, both for sows that received the ID and the SD. We measured a significant reduction in total lesion score on the claws of sows on SD, compared to that of sows on the ID. These findings are in line with the previously documented beneficial effect of chelated Zn, Cu and Mn on the health status of the sow’s claws by decreasing the incidence and severity of lesions on the majority of its anatomical sites [29, 30, 51]. The reported association between the clinical score of sow’s claw with laminitic changes [5] renders the investigation of the effect of chelated minerals at the histologic level and in the quality of the produced hoof horn crucial for understanding the mode of action of their beneficial impact on sows’ hoof clinical health.

Trace minerals, especially zinc, copper and manganese, have an important role in maintaining horn integrity since they have an instrumental function in the process of keratinization [8, 52]. Specifically, the benefit to hoof keratinization of adequate Zn, Cu and Mn supplementation is related with the reinforcement of the epidermal junctions with the corium, making them less susceptible to separation or breakdown, and ultimately the improved integrity of the epithelial tissue [15]. It is theorized that they are capable of enhancing the wound healing process and that they may play an important role in minimizing laminitis through their roles in immune function, the production of horn tissue, and maintenance of epithelial and connective tissue [15, 52]. The unique coordination chemistry of organic compounds permits the formation of highly soluble, chemically stable products that resist digestion and interaction with antagonists in the gut [53, 54]. Therefore, the use of chelated minerals could enhance their absorption and utilization in the body [25, 28], probably due to increased bioavailability [55, 56], and thus the integrity of keratinized tissues [15, 57]. According to studies conducted in bovine, there was a positive effect of chelated minerals on hoof horn quality assessed by histologic and morphometric examination. Specifically, Stern et al. [19] reported an improvement in the microscopic horn quality of the coronary band and tensile strength, when beef calves were fed with organic zinc in a corn and grass silage based diet compared to calves fed with zinc oxide. In addition, Kessler et al. [58] suggested that, even though not significantly improved, bulls offered proteinated zinc tended to have a better histologic score and higher tensile strength of claw horn compared to other zinc sources.

In the initial descriptive statistical analysis, we found that the proportion of culled sows with at least one claw with lamellar hyperplasia of the epidermis was significantly lower for the sows that received the SD compared to those that received the ID. However, in the multivariable analysis, the probability of occurrence of hyperplasia, when adjusting for foot and toe location and the respective total clinical score, did not differ between claws of sows that received the SD and those that received the ID. An inherent defect of the implemented study design was that histologic evaluation of sows’ claws could be performed only once on the same animal, depriving the ability of a within-subject analysis [30]. Therefore, the appraisal of the possible effect of the nutritional intervention could not follow-up the longitudinal course of lamellar hyperplasia. Thus, it was not possible to discern between already existing (prior to the administration of the SD) or recently (during the administration of the SD) developed hyperplastic changes, and between improvement or deterioration of existing ones. Therefore, the lack of a significant difference in the probability of occurrence of hyperplasia between the sows that received the two mineral sources may possibly be attributed to pre-existing changes of hyperplasia that were either irreversible or in order to regress may demand more prolonged use and/or higher substitution rate of inorganic Zn, Cu and Mn with their chelated form. We observed that lamellar hyperplasia could be characterized as severe, in animals in the ID group, with several suprabasal cell layers along the cornified part of the epidermal lamellae, whereas it could be characterized as milder in samples from sows in the SD group, with fewer suprabasal cell layers observed (data not shown).

We also investigated the effect of the two diet mineral sources on the total number of histologic changes recorded when the sows were culled. Our findings indicated that claws of sows on the SD were more likely to have none than at least one histologic change indicative of laminitis, compared to those of the sows on the ID. The reduced frequency of laminitic changes may ensure and/or enhance the adequate and unobstructed supply of oxygen and nutrients needed for the production of high quality hoof horn [8]. The beneficial effect of chelated minerals, at the histologic level, may complement and explain their beneficial impact in the reduction of frequency and severity of clinically evident claw lesions [30]. Chelated minerals, probably, provide a healthier histologic profile in the claw’s dermis and epidermis, through preventing mechanisms or healing properties [15]. Therefore, the progress of existing changes could be delayed or the development of others could be averted, leading to the improvement of the macroscopically observed claw lesion status.

In agreement with previous studies in bovine [36], equine [35] and swine [5], three zones of morphologically different tubules were also identified in the present study. Morphometric assessment of the wall horn revealed evidence, compatible with higher hoof horn quality in the claws of sows fed with the SD compared to sows which received the ID. The improved appearance of the horn architecture, in the light microscopy, was manifested especially in the form of smaller tubules, as increased diameter of the tubules, has been implicated in the genesis of qualitatively inferior horn [59–62]. Specifically, tubules in hoof horn from sows that received the SD had smaller vertical and horizontal diameters, and only few of them were filled with blood components (plasma and hemosiderin). Varagka et al. [5] reported that the vertical and horizontal diameter of the tubules was increasing with increasing severity of wall lesions and claws with severe wall lesions had less tubular density compared to those with no lesions. In the present study, tubular density was higher in the outer zone (zone A) of the wall horn of claws from sows that received the SD compared to that of claws from sows on the ID. As suggested by Gunther et al. [11] and Geyer and Tagwerker [12] for cattle and pig hoof, respectively, “hardness” is related to tubule density. Moreover, measures of hoof hardness are negatively correlated with lameness and lesion severity scores in cattle [63, 64]. According to relevant studies in equine hoof wall, tubules are considered to serve, primarily, a mechanical role, providing reinforcement in a composite structure, offering resistance against fractures and cracks and crack-stopping properties [37]. The above findings indicate a comparative advantage of the hoof horn of claws of sows on the SD in terms of quality measures.

Tubule density in zone B of claw wall horn did not differ among sows that received the SD compared to those of sows that received the ID, whereas it was significantly lower in zone C (inner zone) of claws from the former compared to the latter sows. Tubules in the inner zone have been described as indicators of laminitis in both pigs [40] and horses [41], since this region is active in tubular production from the epidermal lamellae [42, 43]. The above finding when co-evaluated with, the higher odds for either no or less histologic changes indicative of laminitis (i.e., hyperplasia, white blood cells, hyperemia, hemorrhage, edema, necrosis) [5, 32, 65–67] in claws’ dermis and epidermis of the sows on the SD, may suggest a lower incidence or severity of existing or in progress cases of laminitis in the claws of the respective sows.

One may question our use of a “before-and-after” study design in comparison to forming and following-up two groups of animals on ID and SD. However, there are issues that make the applied design the only practical. In these three herds we conducted a larger study whose results have been presented in a series of papers. During the course of the larger study, the farmers became aware of the frequency and severity of sow hoof lesions and their negative effect on productivity [3, 5, 49]. Also, they became aware of the clinical improvement of hoof lesion severity scores and the improvement in reproductive parameters after partially substituting inorganic with organic minerals [30] [68]. It was, therefore, not only unethical to the animals in the control group on the grounds of depriving an acceptable “treatment” for hoof lesions and associated lameness but also, practically speaking, not acceptable by the farmers to form and follow-up two groups with animals receiving different diets. Moreover, during the course of the herewith reported study, there were no alterations in known significant risk factors for sow hoof lesions such as flooring, group housing and feeding, or feed formulation standards in these herds.

## Conclusions

In conclusion, partial substitution of inorganic Zn, Cu and Mn provided in sows’ diets with their chelated complexes, had a beneficial effect, at least under the conditions of this study, on the overall health and quality of the sows’ claws, assessed by macroscopic, histologic and morphometric examination. Therefore, the effect of chelated Zn, Cu and Mn in the reduction of claw lesion severity and frequency could be regarded as the clinically observable result of their role in the improvement of the normal, healthy histologic structure of claw’s dermis and epidermis and the subsequent enhancement of the sows’ hoof horn quality.

## Abbreviations

ID inorganic diet; ME, metabolizable energy; SD, supplemented diet
